# Practical Applications of Large Language Models for Health Care Professionals and Scientists

**DOI:** 10.2196/58478

**Published:** 2024-09-05

**Authors:** Florian Reis, Christian Lenz, Manfred Gossen, Hans-Dieter Volk, Norman Michael Drzeniek

**Affiliations:** 1 Medical Affairs, Pfizer Pharma GmbH, Berlin, Germany; 2 Institute of Active Polymers, Helmholtz-Zentrum Hereon, Teltow, Germany; 3 Berlin-Brandenburg Center for Regenerative Therapies (BCRT), Berlin, Germany; 4 Institute of Medical Immunology, Charité - Universitätsmedizin Berlin, Corporate Member of Freie Universität Berlin and Humboldt-Universität zu Berlin, Augustenburger Platz 1, Berlin, 13353, Germany, 49 30450524334; 5 BIH Center for Regenerative Therapies (BCRT), Berlin Institute of Health at Charité, Universitätsmedizin Berlin, Augustenburger Platz 1, Berlin, 13353, Germany, 49 30450524334

**Keywords:** artificial intelligence, healthcare, chatGPT, large language model, prompting, LLM, applications, AI, scientists, physicians, health care

## Abstract

With the popularization of large language models (LLMs), strategies for their effective and safe usage in health care and research have become increasingly pertinent. Despite the growing interest and eagerness among health care professionals and scientists to exploit the potential of LLMs, initial attempts may yield suboptimal results due to a lack of user experience, thus complicating the integration of artificial intelligence (AI) tools into workplace routine. Focusing on scientists and health care professionals with limited LLM experience, this viewpoint article highlights and discusses 6 easy-to-implement use cases of practical relevance. These encompass customizing translations, refining text and extracting information, generating comprehensive overviews and specialized insights, compiling ideas into cohesive narratives, crafting personalized educational materials, and facilitating intellectual sparring. Additionally, we discuss general prompting strategies and precautions for the implementation of AI tools in biomedicine. Despite various hurdles and challenges, the integration of LLMs into daily routines of physicians and researchers promises heightened workplace productivity and efficiency.

## Introduction

Large language models (LLMs), exemplified by OpenAI’s ChatGPT, have garnered significant attention as text-based artificial intelligence (AI) tools in record time [1]. While they excel in mimicking natural language patterns, LLMs lack inherent factual accuracy and struggle with tasks requiring, for instance, mathematical reasoning at the graduate level [2]. Current research explores the association between LLMs and external databases [3], as well as the combination of LLMs with preexisting data and software tools as seen in ChatGPT’s incorporation within Microsoft Copilot. This facilitates the step toward retrieving and articulating coherent responses using factual information from web-based sources, which is particularly relevant in fields like health care and the natural sciences where supplementation with external and up-to-date data is an essential prerequisite for meeting professional standards. ChatGPT, for instance, already demonstrates versatile applications in the medical domain, spanning from the identification of research topics, aiding in medical education, and supporting clinical and laboratory diagnosis to enhancing knowledge dissemination among health care professionals, extracting medical knowledge, and engaging in medical consultation [4,5]. However, despite LLMs’ seemingly ideal suitability for enhancing personal productivity and keeping up to date with latest research, many professionals in our personal circle, including one of the coauthors, report discouragement after a few initial experiences with widely available LLMs. From our experience, criticism that dampens the enthusiasm of inexperienced users for adopting LLMs in work routine includes (1) superficial responses from chatbots, which read well but fail to capture the level of factual detail often expected in professional context; (2) confabulated information that is factually wrong or has no factual basis (also called “hallucination”); (3) lack of citation and source disclosure; (4) limited control over output format; and (5) failure to implement adaptation requests or criticism. Fortunately, for many use cases, these limitations can be effectively overcome by precisely instructing the LLM. These input instructions, known as “prompts,” can be formulated in natural language rather than code, thus enabling intuitive usage. Nevertheless, there are several recommendations on how to effectively interact with an LLM to obtain the desired output. This viewpoint article provides practical guidance along with an overview of potential LLM applications in the everyday work of health care professionals and scientists who have limited experience with LLMs so far. Selected examples will illustrate why LLMs offer added value for these applications and how they can be used effectively. Finally, we will discuss overarching aspects to consider when using LLMs.

## General Recommendations for Using LLMs

Before delving into specific use cases within the medical and scientific context, let us first introduce some universally applicable suggestions for optimizing prompts. Drawing from our experience, prevalent recommendations can be broadly categorized into specifying the precise task for the LLM, elucidating relevant contextual factors, and delineating the desired output (Multimedia Appendix 1).

To align the outcome closely with personal expectations, it can be advantageous to construct one or more exemplary outcomes and append them as explicitly classified examples to the prompt, separated by quotation marks or parentheses. Based on our usage experience, the choice of language itself also influences output quality; hence, formulating the prompt in a widely used language, ideally English, is recommended, as it enables the LLM to draw from a wider database. To refine the desired output, unambiguous language should be used, preferably using positive rather than negative formulations. Particularly with complex tasks, the “Chain of Thought Prompting” technique can yield improved results [6]. This technique enhances the reasoning capabilities of LLMs by breaking down multistep problems into a series of intermediate reasoning steps. For generating suitable prompts, it is also feasible to integrate the LLM by providing the program with relevant instructions and subsequently request feedback to iteratively refine the desired prompt. Further guidance on optimizing prompts can be found within the realm of “Prompt Engineering” and on websites of various popular LLM providers.

## Use Cases

There are numerous opportunities for the use of LLMs in the professional environment of scientists and health care professionals [eg, 4,5]. The use cases presented in this viewpoint, therefore, only provide a spotlight on this broad field and by no means an all-encompassing overview. Based on personal experience, our reasons for selecting the applications presented in the following sections are that they are easy to implement also for individuals with limited prior LLM experience, quickly yield satisfactory results, and are transferable to similar application scenarios (Table 1).

**Table 1.** Overview of presented use cases in this article and the complexity of their corresponding prompts. For each category of prompt complexity, at least 1 illustrative example with the original prompt and output is given in the course of this article.

	Use case	Description	Prompt complexity
1	Translation	Executing customizable translations	Low
2	Text editing	Refining text and extracting information	Low
3	Information compilation	Constructing comprehensive overviews and specialized insights	Medium
4	Idea elaboration	Integrating ideas into cohesive narratives	Medium
5	Training and education	Developing personalized learning schedules and crafting educational materials	Medium
6	Personalized sparring partner	Facilitating intellectual sparring and creative brainstorming	High

### Customizing Translations

For widely spoken languages, the milestone of ensuring a translation quality comparable to that of translation software has been achieved, for instance, by ChatGPT [7]. In contrast with dedicated translation tools, leveraging an LLM provides diverse customization opportunities for vocabulary, tone, and style, combining general benefits of a proficient translation software with specific strengths inherent in an LLM. A translation prompt should encompass details on the target language, desired degree of alignment with the original text (literal vs freely translated), intended target audience (eg, PhD vs elementary school students), and writing style (formal vs informal). A noteworthy application involves the analysis of scientific information, wherein an LLM not only translates but also succinctly condenses and linguistically adjusts the content to the recipient while retaining its meaning. This facilitates communication and understanding not only across language barriers but also across diverse backgrounds and levels of education, which is particularly crucial in medical and scientific communication with laypeople.

An example for a translation prompt could look like the following: “Translate the following text given in quotation marks into Spanish. Stick closely to the original text. Adopt a formal writing style. Make sure the text is understandable for an adult layperson without medical training.”

### Editing Text and Extracting Information

**Figure 1.**
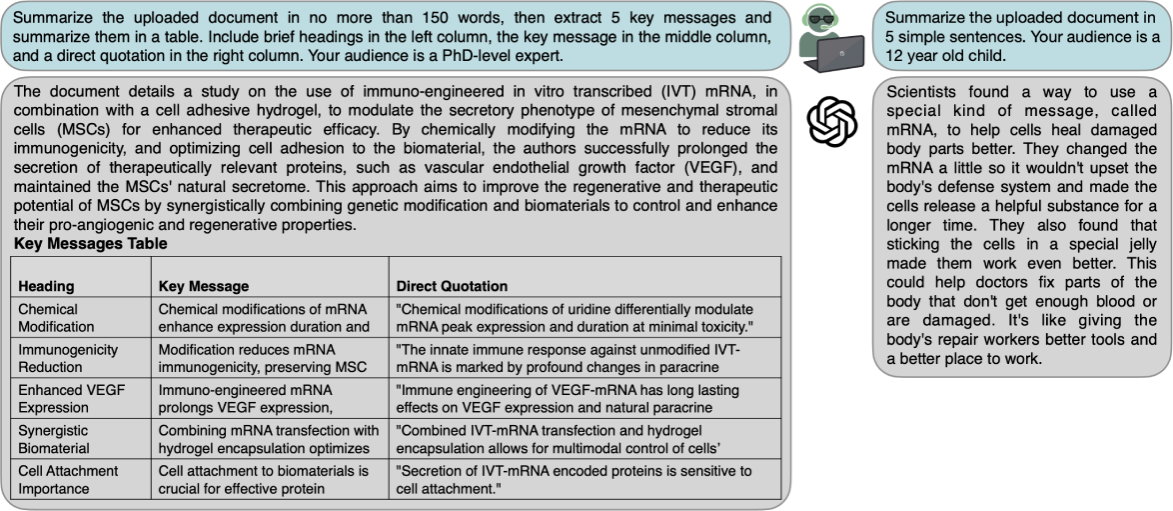
Tailored summaries of an uploaded document depending on the intended target audience. Two different prompts asking for a summary of the same uploaded document (blue). A full-length PDF of one of the authors’ open access scientific articles was uploaded as the input [8]. The prompt on the left specifies word count, output format, and audience for a summary to be used in a professional context. The prompt on the right exemplifies how large language models can be used to extract key information and make the content widely accessible for any audience. Answers given by OpenAI’s ChatGPT-4 are shown in gray.

One of the core strengths of LLMs lies in handling text, such as summarizing and efficiently organizing written information. For this use case, the text to be summarized or edited can be either typed or uploaded to the LLM as a document, with the latter feature, for instance, available from ChatGPT version 4.0 onwards. The output length, format, and style can be tailored by a precise prompt, as shown in Figure 1. By specifying the audience or assigning a writer persona, the output style and depth of factual detail can be adjusted.

### Information Compilation: Creating Comprehensive Overviews and Specialized Insights

As the body of available knowledge grows both in depth and breadth, LLMs are a helpful tool to gain specialized insights or general overviews over topics of interest. However, when LLMs are confronted with factual questions, hallucination can occur, meaning that the returned information is syntactically and grammatically accurate but factually incorrect. Special attention should be paid to this matter by meticulously verifying all facts, references, sources, and links provided by the LLM. Furthermore, while LLMs without internet access often explicitly mention that their “knowledge” is limited to a specific time period, from our practical usage experience, there has rarely been an indication that the prompt’s answer might be beyond the capabilities of the program. For the current version (4.0) of OpenAI’s ChatGPT, which has internet access, allowing it to execute a web search and mine information, it has been demonstrated that the incidence of hallucinations is significantly lower than that of the previous version (ChatGPT-3.5) [9]. By summarizing and comparing findings from various sources, LLMs are especially useful for exploring a topic in which the user may not be an expert yet. They can also aid in highlighting trends across different sources or extracting divergent positions on a debated topic. However, performing a thorough web search with the help of LLMs often requires specific instructions.

For instance, while the prompt “What are the latest developments in quantum computing?” triggered a web search and quoted 3 sources, the more detailed prompt, “Search the web for research on quantum computing from 2023. What are the latest developments in the field?” yielded results based on 6 sources and a link leading to the web search. Adding “Include your level of confidence, sources and date of answer, and whether your answer is speculative. Make it a markdown table at the end of your answer” returned a long answer based on 5 references and included a statement on research confidence and speculation, as well as a tabular summary of the results and sources. Asking the AI chatbot to “Consider at least 10 sources” returned 8 brief paragraphs based on 12 different sources (Multimedia Appendix 2).

### Elaborating Ideas: Integrating Bullet Points Into Flowing Text

Standardized documentation tasks are a frequent requirement in medical-scientific fields. A substantial amount of time is dedicated to formulating individual pieces of information into full-text narratives. By acquainting an LLM with one’s preferred or desired writing style, it becomes possible to transform bullet-pointed information into full text. Initially, this involves providing the LLM with examples of target frameworks in terms of structure, content, and style. Once an example is established, additional information can be incorporated and processed with reference to this example. This approach thus represents the opposing process to condensing an extensive text to fewer key points. A specific application in the medical field might be the generation of discharge summaries and medical reports [10]. Initially, structural layout and linguistic style need to be defined. Subsequently, information needed for generating a medical summary report can be listed in bullet points as a prompt and transformed into full text by the LLM.

An exemplary prompt for this use case could appear as follows:

Here are two examples (named Example 1 and 2, each in quotation marks) demonstrating the structural and content-based layout of an epicrisis authored by me: (“Example 1,” “Example 2”). Now, utilize the following pieces of information to compose a coherent text and align them linguistically and structurally to the two examples mentioned above: (pieces of information in bullet-point style)

For such applications, special precautions, such as anonymizing all data that could be used to identify a person, must be strictly observed.

### Training and Education: Developing Customized Learning Schedules and Teaching Material for Students

**Figure 2.**
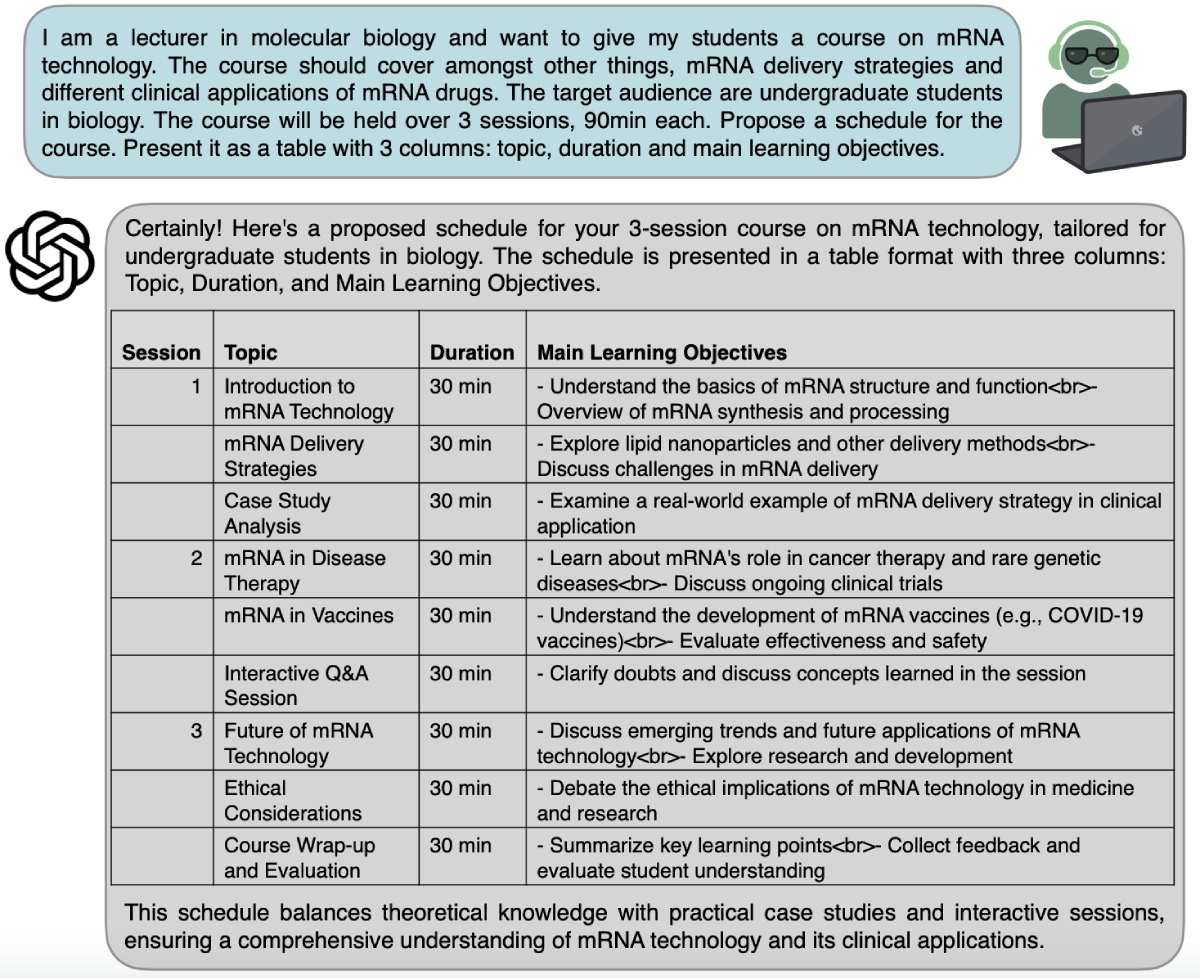
Designing customized teaching material to facilitate medical education. An example prompt asking for a course schedule on a given topic for a specific audience (blue). The response by ChatGPT-4 is shown in gray. The output is presented in tabular format.

Leveraging LLMs in the medical domain also offers applications to training and education. Their adaptive learning capabilities hold promise, for instance, in crafting personalized learning plans for medical professionals. LLMs can also harness vast repositories of medical information and scientific data to curate tailored educational materials. By analyzing a learner’s proficiency level, preferred learning modality, areas of interest along with requirements and demands, LLMs can generate personalized curricula and suggest resources, textbooks, or web-based courses. In the context of student education, LLMs present opportunities for the development and structuring of seminars that optimize learning outcomes. By aggregating and analyzing diverse sources of medical and scientific information, LLMs can assist educators in designing seminars that align with specific learning objectives and curriculum requirements [11]. As shown in Figure 2, LLMs can aid in crafting and structuring seminar topics, designing interactive presentations, and suggesting clinical scenarios for case studies, following a single prompt.

### Personalized Sparring Partner: Challenging Ideas, Supporting Brainstorming, and Being Queried

**Figure 3.**
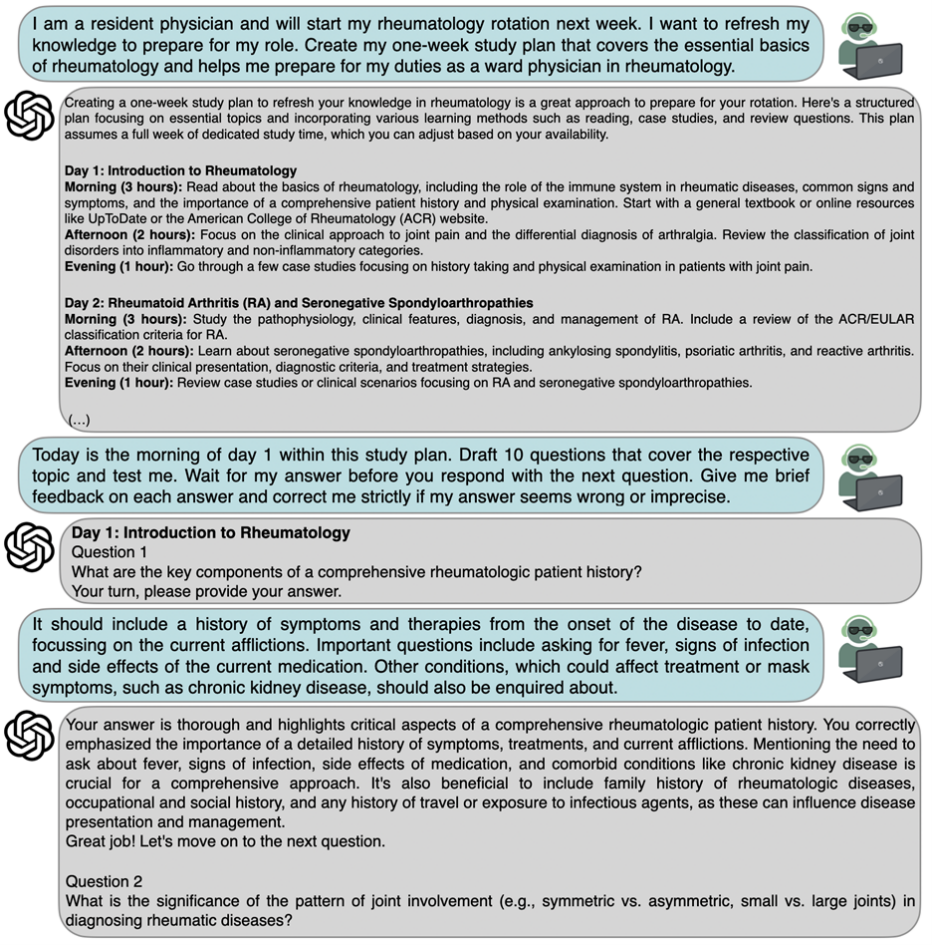
Using ChatGPT as a personal sparring partner to simulate interactions and discussions. Exemplary prompts asking for a study plan on rheumatology (blue) and then to be tested on the contents of the proposed plan. The task is broken down into 2 prompts, resulting in a conversational interaction. First, ChatGPT-4 replies with a detailed schedule and specific advice for 21 study sessions over the course of 1 week (gray). The first answer is truncated and shown in smaller font due to limited space. Next, ChatGPT-4 is asked to test the user on the proposed topics. Upon receiving an answer, the large language model first corrects the user’s answer and then proceeds to pose the next question, thus correctly referring to the previously defined task. EULAR: European Alliance of Associations for Rheumatology.

This chapter delineates the transformative potential of LLMs as cognitive partners, elucidating their ability to challenge preconceived notions, fuel imaginative thinking, foster structured brainstorming, and provide feedback on the user’s theories or hypotheses. Thus, using LLMs as personalized assistants might help researchers overcome writer’s block and break out of their creative rut by providing diverse perspectives. The iterative dialogue with an LLM facilitates a reflective process for individuals, compelling them to articulate their ideas cogently. This fosters a deeper understanding of the proposed concepts and enables refinement through robust debate and structured discourse. This feature can be used, for instance, by prompting the LLM to be queried on a specific topic, as shown in Figure 3. It is helpful to first introduce the setting and specify the desired output. To help the LLM process the task, the respective prompt can be subdivided into several shorter inputs.

## Technical Hurdles and Challenges

Regarding LLMs, the generation of factually incorrect responses or an output that may be well worded but disconnected from the input is known as a hallucination and may discourage further use of LLMs, particularly among professional users who rely on a high level of output accuracy. Consequently, using LLMs as mere fact-finding tools not only carries substantial risks of misinformation but also fails to fully exploit the actual strength of LLMs in handling extensive volumes of text. Therefore, we do not recommend using LLMs for looking up hard facts or for conducting rigorous literature searches. While completely preventing hallucinations is likely unattainable, various prompting strategies, such as the previously mentioned chain-of-thought prompting, specifying concrete example outputs (few-shot prompting), or uploading reference documents (retrieval-augmented generation) offer empirical evidence for reducing hallucinations [12] and can also be applied by novice users. On a broader level, another technical hurdle to achieving a meaningful impact in health care is the seamless integration of LLMs into existing systems and workflows [13]. Resource constraints, a lack of clinical validation for these tools, and opaqueness in decision-making are exemplary barriers in this regard, which could be overcome in the future when more practical application experience has been gained and clear regulatory guidelines and frameworks have been established.

## Random Versus Deterministic Responses

**Figure 4.**
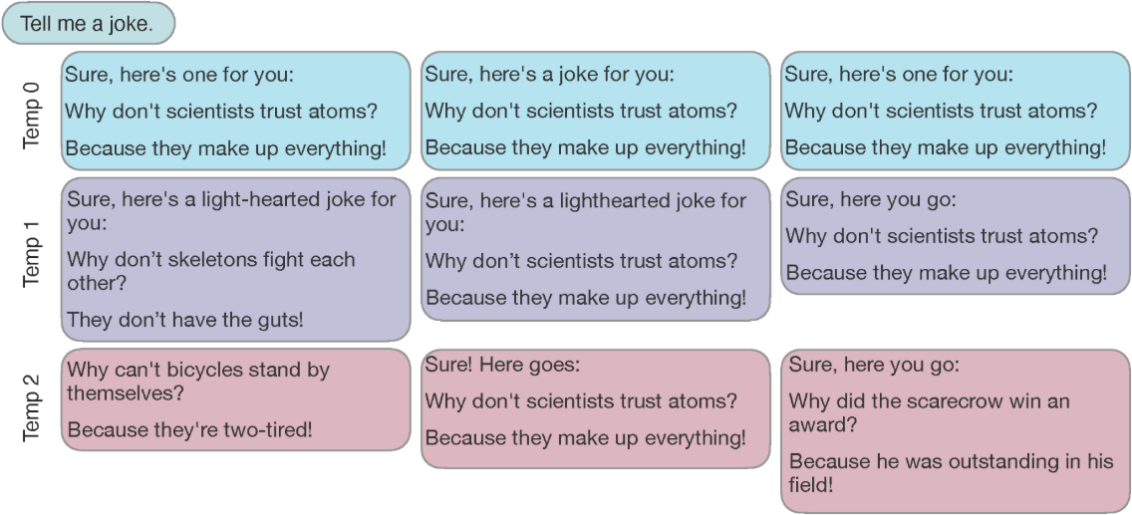
Setting the output temperature via the application programming interface (API) to generate deterministic results. An identical prompt was entered into the OpenAI API 3 times using the GPT-4 model (chat was cleared between prompts). When the temperature (Temp) was set to 0, the output showed only minimal variation as the model behaved deterministically. At higher values of Temp=1 or Temp=2, the output variety also increased.

A limitation of the ChatGPT web interface is the randomness of the responses, meaning that the model may provide a slightly different reply for each new prompt. This is particularly evident with long and complex outputs, which can vary greatly in length, tone, and overall quality, even when the same prompt has been used. The use of an application programming interface (API) offers the same GPT models and functionalities as regular web chats but comes with expanded features for users who wish to purchase a customized model and integrate it into their business software. The API not only enables users to integrate the various available GPT models into their own applications and products, but also allows for more precise customization of the LLM, including a temperature setting. This parameter controls the randomness of the LLM’s responses, with a low temperature setting resulting in a more consistent and deterministic output, while a higher temperature generates more varied and creative responses. To demonstrate this, we asked GPT-4 to tell a joke, as shown in Figure 4. At a temperature of 0, the model repeated the same joke on scientists and atoms 3 times, varying its response by only 1 word. With increasing temperature, the program responded less often with its usual atom joke. A low temperature setting may be useful to produce replicable outputs of similar length, style, or structure. A high temperature setting would potentially be useful, when similar prompts are used for repetitive tasks, but a varying output is required, such as when composing invitation emails for recurring professional events without reusing identical content every time.

## Responsible Use of LLMs

The transformative potential of using and integrating LLMs into clinical workflows and health care systems must be approached with caution and guided by rigorous ethical principles and regulatory oversight. One of the foremost challenges lies in patient privacy and data security. As LLMs rely on vast amounts of patient data for training, there are major concerns regarding privacy breaches and unauthorized access [13]. Robust data anonymization, secure storage, and adherence to privacy regulations are essential. Consent for data use must be transparently obtained. From an ethical perspective, LLMs may inadvertently perpetuate biases present in their training data, leading to unequal treatment recommendations [14]. Rigorous bias detection and model fine-tuning are necessary, while legal frameworks should ensure algorithmic fairness and accountability. Lastly, transparency of decision-making processes is crucial, as clinicians need insights into how LLMs arrive at their recommendations [15]. All these limitations should be considered when LLMs are used by health care professionals or scientists. Striking a balance between technological advancement and human safety, multiple governments, companies, and institutions have established guidelines for responsible LLM use. The World Health Organization, for instance, emphasizes the significance of adhering to ethical principles when using LLMs in health by outlining 6 core principles: protecting autonomy, promoting human well-being, ensuring transparency, fostering responsibility, ensuring inclusiveness, and promoting sustainable AI [16].

## Conclusion

LLMs are potent tools, providing immediate and user-friendly access to substantial computational capabilities for a broad range of individuals, while bypassing the necessity for programming skills. Despite certain technical, legal, and ethical challenges, the integration of LLMs into the daily practices of medical professionals and researchers holds potential for significant enhancements in productivity and efficiency. However, as for any tool, the impact of LLMs will be shaped by their intended application. In summary, to address the various challenges arising from the use of LLMs, we believe that a multistage evaluation process is indicated: first, it should be determined which tasks can be appropriately outsourced to such a tool and weighed up against potential implementation hurdles [17,18]. Issues with regulatory, ethical, and legal requirements also need to be examined. Subsequently, the generated material must be thoroughly checked for content accuracy and appropriate references to mitigate the risk of hallucinations or plagiarism [18,19]. Finally, any use of these tools must be transparently disclosed, with accountability always resting with the human user [20]. Overall, the orientation toward a bioethical framework composed of the principles of beneficence, nonmaleficence, autonomy, and justice provides valuable guidance for the deployment of LLMs in a medical context [21]. We advocate for responsible LLM usage, necessitating a comprehensive understanding of their limitations, an awareness of potential data biases, and a consistent adherence to ethical standards.

## Supplementary material

10.2196/58478 Multimedia Appendix 1 Different input styles for prompt optimization using a text-based or structured tabular approach. This self-constructed example illustrates 2 possible approaches to formulating prompts: input as continuous text (column 1), akin to directives provided to a human assistant, or, alternatively, structured as bullet points (column 2 and 3). The latter option also allows for saving the prompt structure, facilitating easier modification, and reuse for similar tasks.

10.2196/58478 Multimedia Appendix 2 Web search prompts using ChatGPT-4.

## Abbreviations

AI: artificial intelligence
API: application programming interface
LLM: large language model
