# Supplementary material for: Practical Applications of Large Language Models for Health Care Professionals and Scientists
Source: JMIR Med Inform. 2024 Sep 5;12:e58478. doi: 10.2196/58478 (PMC11391657; doi:10.2196/58478)
Supplement: Multimedia Appendix 1 [file medinform-v12-e58478-s001.docx]

| **Natural Language Prompt** | **Structured Prompt** | |
| --- | --- | --- |
|  | **Section** | **Input** |
| Write a scientific report on the applications of Large Language Models (LLMs) in the context of diagnosing and treating the most globally relevant rare diseases. | ***Task*** | Write a scientific report. |
|  | ***Topic*** | Applications of Large Language Models (LLMs) in the context of diagnosing and treating the most globally relevant rare diseases. |
| The report should encompass a thorough analysis of the current utilization of LLMs, their effectiveness, and potential impacts on the treatment of rare diseases. A separate section should highlight potential risks and limitations associated with implementing LLMs in these areas and present solutions in the form of a table with two columns (Column 1: Potential risks and limitations; Column 2: Possible solutions). Conclude with a paragraph outlining potential theories regarding the future development and utilization of LLMs in medical diagnosis and treatment of rare diseases, providing explanations. | ***Content*** | Thorough analysis of the current utilization of LLMs, their effectiveness, and potential impacts on the treatment of rare diseases.  Separate section should highlight potential risks and limitations associated with implementing LLMs in these areas.  Create a table with two columns (Column 1: Potential risks and limitations; Column 2: Possible solutions).  Conclude with a paragraph outlining potential theories regarding the future development and utilization of LLMs in medical diagnosis and treatment of rare diseases, providing explanations. |
| Keep the word count within 1000 words. | ***Language*** | Scientific English |
| Compose your report in scientific English using an objective tone. | ***Tone*** | Objective |
|  | ***Length*** | 1000 words or less |
| Utilize information from scientifically sound sources. Proceed step by step: first search the web for appropriate sources. Then write a report which considers all sources. | ***Sources*** | Information from scientifically sound sources |
|  | ***Process*** | First search the web for appropriate sources. Then write a report which considers all sources. |
| You are an expert in rare diseases at a university hospital. | ***Persona*** | Expert in rare diseases at a university hospital |
| Your report will be presented to the research staff of your institute to provide them with an overview of this subject. | ***Recipient*** | Institute research staff |
|  | ***Aim*** | Provide overview |

Table 1: **Different input styles for prompt optimization.** *This self-constructed example illustrates two possible approaches to formulating prompts: input as continuous text (column 1), akin to directives provided to a human assistant, or, alternatively, structured as bullet points (column 2 and 3). The latter option also allows for saving the prompt structure, facilitating easier modification, and reuse for similar tasks.*
