# Supplementary material for: Practical Applications of Large Language Models for Health Care Professionals and Scientists
Source: JMIR Med Inform. 2024 Sep 5;12:e58478. doi: 10.2196/58478 (PMC11391657; doi:10.2196/58478)
Supplement: Multimedia Appendix 2 [file medinform-v12-e58478-s002.docx]

SUPPLEMENTARY MATERIAL

**Practical Applications of Large Language Models for Healthcare Professionals and Scientists**

*Florian Reis^1^, MD, Christian Lenz^1^, MD, Manfred Gossen^2^, PhD, Hans-Dieter Volk^3^, MD/PhD,*

*Norman Michael Drzeniek^3^, MD/PhD*

^1 Pfizer Pharma GmbH, Friedrichstraße 110, 10117, Berlin, Germany.^

^2 Institute of Active Polymers, Helmholtz-Zentrum Hereon, Kantstraße 55, 14513 Teltow, Germany; Berlin-Brandenburg Center for Regenerative Therapies (BCRT), Augustenburger Platz 1, 13353 Berlin, Germany.^

^3 Charité - Universitätsmedizin Berlin, Corporate Member of Freie Universität Berlin and Humboldt-Universität zu Berlin, Institute of Medical Immunology, Augustenburger Platz 1, 13353, Berlin, Germany; Berlin Institute of Health at Charité - Universitätsmedizin Berlin, BIH Center for Regenerative Therapies (BCRT), Föhrer Straße 15, 13353, Berlin, Germany.^

**Table of Contents:**

Supplementary Information 1. Example of web search prompts using ChatGPT 4.0 ........................................... 1

**Supplementary Information 1: Example of web search prompts using ChatGPT 4.0:**

The four example conversations shown below were directly copied from the ChatGPT web interface. Functioning hyperlinks to sources were automatically replaced by placeholders in bold brackets:

User: What are the latest developments in quantum computing?

ChatGPT: The latest developments in quantum computing as of 2023 are marked by significant advancements in both hardware and software, with major contributions from IBM and other key players in the field.

1. **IBM's Quantum Computing Milestones**:

- IBM has unveiled the IBM Quantum System Two, the company’s first modular quantum computer, which forms the cornerstone of its quantum-centric supercomputing architecture. This system began operations with three IBM Heron processors and supporting control electronics【6†source】.

- The IBM Quantum Heron processor, also released in 2023, represents IBM's best performing quantum processor to date. It offers up to a five-fold improvement in error reduction compared to previous architectures【6†source】.

- IBM has extended its IBM Quantum Development Roadmap to 2033, aiming to significantly advance the quality of gate operations, which would increase the size of quantum circuits that can be run【6†source】.

- IBM’s breakthrough in quantum computing, demonstrated with the IBM Quantum 'Eagle' processor, showed that quantum computers could produce accurate results at a scale of 100+ qubits, surpassing leading classical approaches in specific simulations【9†source】.

2. **Shift in Focus towards Error Correction and Fault Tolerance**:

- The industry is moving away from the "noisy intermediate-scale quantum" (NISQ) idea towards more robust quantum computing methods. This shift involves using some qubits to correct errors in others, as demonstrated by Google Quantum AI and Quantinuum【7†source】.

- IBM has been exploring methods to mitigate noise by characterizing the error-inducing noise in its machines and programming a way to subtract it, akin to noise-canceling headphones【7†source】.

- IonQ, working on trapped-ion quantum computers, is implementing similar noise mitigation techniques to improve their error rates【7†source】.

3. **Software Development and Hybrid Quantum Computing**:

- There's a growing need for more advanced quantum programming tools. Companies like Horizon Quantum Computing and Algorithmiq are developing programming tools that allow for more flexible computation routines【7†source】.

- Hybrid quantum computing, which combines quantum and classical algorithms, is a key area of focus. Algorithmiq’s drug discovery platform, Aurora, is an example of this approach, aiming to achieve a useful quantum advantage in 2023【7†source】.

4. **Global Research and Industry Applications**:

- IBM’s advancements in quantum computing are being leveraged by various industries and research institutions. In healthcare and life sciences, organizations like Cleveland Clinic and Moderna are exploring applications in molecular discovery and patient risk prediction models. In high energy physics, institutions like CERN are investigating quantum calculations for particle collision events. The materials sector, including companies like Boeing and ExxonMobil, is exploring methods for materials simulation. In the field of optimization, collaboration is ongoing to identify problems best suited for quantum advantage in areas like sustainability and finance【9†source】.

These developments indicate that quantum computing is transitioning from a primarily theoretical and experimental phase to one where practical applications and utility-scale explorations are becoming increasingly feasible. The focus on error correction, fault tolerance, and hybrid computing strategies suggests a maturing of the field, poised to address more complex and real-world problems.

....................................................................................................................................................................................

User: Search the web for research on quantum computing from 2023. What are the latest developments in the field?

ChatGPT: 2023 has been a significant year for advancements in quantum computing, marked by several notable developments that have pushed the boundaries of the field. Here's an overview of the key breakthroughs:

1. **Logical Quantum Processor by Harvard Researchers**: A team at Harvard, led by Mikhail Lukin, has created the first programmable, logical quantum processor. This processor can encode up to 48 logical qubits and execute hundreds of logical gate operations, a considerable improvement over previous efforts. This achievement is a major step towards reliable, scalable quantum computing, marking the beginning of early fault-tolerant quantum computation【6†source】.

2. **Enhanced Qubit Coherence Time by DOE/Argonne National Laboratory**: Researchers have significantly advanced quantum computing architecture by prolonging the coherence time of their single-electron qubit to an impressive 0.1 milliseconds. Coherence time is crucial as it measures how long a qubit can perform operations or run algorithms before losing its information, and extending this time is vital for practical quantum computing applications【7†source】.

3. **IBM's 1,000-Qubit Quantum Chip**: IBM has unveiled a quantum processor with more than 1,000 qubits, named Heron. This development is part of IBM's roadmap to achieve useful computations with quantum computers by the end of the decade, such as simulating the workings of catalyst molecules, which would be a significant stride in material science and other fields【8†source】.

4. **IBM Quantum Computer's Performance Beyond Classical Supercomputing**: IBM's Quantum 'Eagle' processor, composed of 127 superconducting qubits, has been shown to outperform leading classical simulations in modeling physical systems, such as the dynamics of spins in materials. This milestone suggests that quantum computers are now entering a new era of utility, where they can tackle problems that are extremely difficult or impossible for classical systems【9†source】.

5. **High-Fidelity Quantum Operations with Fluxonium Qubits at MIT**: Researchers have found a way to use fluxonium qubits, which have longer lifespans or coherence times than traditional transmon qubits, in an architecture supporting robust, high-fidelity gates. Fluxonium qubits achieved coherence times more than ten times longer than transmon qubits. This improvement is vital for quantum error correction and the development of larger-scale, more reliable quantum computers【10†source】.

6. **DARPA-Funded Research on Quantum Circuits**: DARPA-funded research has led to the creation of the first-ever quantum circuit with logical quantum bits (qubits). This discovery could accelerate the development of fault-tolerant quantum computers, a crucial aspect of making quantum computing practically viable【11†source】.

These developments collectively represent significant progress in overcoming the challenges of quantum computing, such as increasing qubit stability and coherence, error correction, and the practical utility of quantum systems in solving complex problems. The advancements in 2023 pave the way for more practical and scalable quantum computing solutions in the near future.

....................................................................................................................................................................................

User: Search the web for research on quantum computing from 2023. What are the latest developments in the field? Include your level of confidence, sources and date of answer, and whether your answer is speculative. Make it a markdown table at the end of your answer.

ChatGPT: The research and developments in quantum computing in 2023 have been marked by several significant advancements, primarily driven by major players in the field such as IBM. Here's a detailed overview of the latest developments:

1. **IBM Quantum Developments**:

- IBM unveiled the 'IBM Quantum Heron', their best performing quantum processor to date, at the IBM Quantum Summit 2023. This processor features a newly built architecture that offers up to five-fold improvement in error reduction compared to previous models.

- The company has extended its IBM Quantum Development Roadmap to 2033, setting new targets to significantly advance the quality of gate operations, which is crucial for realizing the full potential of quantum computing at scale.

- IBM is also focusing on software advancements, particularly with Qiskit 1.0, which aims to simplify quantum software programming. The introduction of Qiskit Patterns and the integration of generative AI into quantum code programming through IBM's enterprise AI platform, watsonx, are steps towards democratizing quantum computing development【6†source】.

2. **Shift in Focus from NISQ to Error Correction and Fault Tolerance**:

- The field is gradually shifting its focus from "noisy intermediate-scale quantum" (NISQ) computers towards more robust error correction and fault tolerance methods. This shift is essential for the development of large-scale quantum computers capable of handling complex computations.

- Companies like Google Quantum AI and Quantinuum have demonstrated the use of qubits in error-correcting ensembles that outperform physical qubits. IBM is also exploring methods to characterize and mitigate error-inducing noise in its machines, similar to noise-canceling technologies.

- IonQ, another major player in the field, is focusing on reducing errors imposed during quantum computations on trapped-ion quantum computers【7†source】.

3. **IBM's Roadmap for Useful Quantum Computations**:

- IBM has laid out a roadmap aiming to achieve useful quantum computations, such as simulating the workings of catalyst molecules, by the end of the decade. This marks a significant shift from the earlier view of quantum computing as a distant dream to a more imminent reality【8†source】.

4. **Advances in Quantum Computing Architecture**:

- A major milestone was achieved in a new quantum computing architecture by researchers at DOE/Argonne National Laboratory. They reported significant advancements in electron charge qubits, achieving a 0.1 millisecond coherence time. This development is crucial as it enhances the stability and potential for quantum computations【9†source】.

5. **DARPA-Funded Quantum Computing Research**:

- DARPA's research efforts have also contributed to significant breakthroughs in quantum computing. However, specific details about these developments were not available in the provided source【10†source】.

These developments indicate a significant shift in the quantum computing landscape, with a focus on improving error rates, developing robust quantum software, and enhancing the scalability and utility of quantum computers. However, it's important to note that while the field is advancing rapidly, many of these developments are still in the experimental or early implementation stages.

### Research Confidence and Speculation

- **Confidence Level**: Moderate-High. The information is sourced from credible and reputable organizations such as IBM, DARPA, and major research laboratories.

- **Sources Used**: IBM Newsroom, MIT Technology Review, Nature.com, ScienceDaily, DARPA.

- **Date of Answer**: December 16, 2023.

- **Speculative Nature**: While the developments are backed by research and announcements from credible entities, the field of quantum computing is inherently experimental and rapidly evolving, making long-term predictions somewhat speculative.

### Table of Developments

| **Development** | **Description** | **Source** |
| --- | --- | --- |
| IBM Quantum Heron Processor | IBM's best performing quantum processor with significant error reduction improvements | IBM Newsroom​​ |
| IBM Quantum Development Roadmap | Extended to 2033 with a focus on improving gate operations and software advancements | IBM Newsroom​​ |
| Shift in Focus to Error Correction | Moving from NISQ to robust error correction and fault tolerance in quantum computing | MIT Technology Review​​ |
| IBM Roadmap for Useful Computations | Aiming to achieve practical quantum computations by the end of the decade | Nature.com​​ |
| New Quantum Computing Architecture | Major milestone by DOE/Argonne National Laboratory in enhancing quantum computing stability | ScienceDaily​​ |
| DARPA-Funded Research | Contributions to breakthroughs in quantum computing, specifics not detailed | DARPA​​ |

....................................................................................................................................................................................

User: Search the web for research on quantum computing from 2023. What are the latest developments in the field? Consider at least 10 sources.

ChatGPT**:** The field of quantum computing has seen remarkable developments in 2023, touching on various aspects from hardware advancements to software and algorithmic innovations, as well as increasing industry and academic collaborations.

1. **Hardware Progress**: A significant breakthrough in 2023 was IBM's release of the first-ever 1,000-qubit quantum chip, a major step in quantum computing capabilities【8†source】. IonQ, a Maryland-based company, has been focusing on trapped-ion quantum computers, working towards reducing errors and improving performance【6†source】.

2. **Software and Algorithms**: The quantum computing community is acknowledging the need for more advanced software and programming tools. Companies like Zapata Computing are working on expanding the quantum software toolbox, which is crucial for future developments【6†source】. This includes efforts to improve quantum algorithm performance against classical computers, as researchers strive to reach a point where quantum algorithms outperform their classical counterparts【13†source】.

3. **Quantum Networks and Communication**: There has been progress in the development of advanced quantum networks, essential for the practical application of quantum computing in various fields【10†source】. This includes the use of quantum repeaters using defects in diamond to interconnect quantum systems, enhancing quantum communication capabilities【11†source】.

4. **Quantum Error Correction and Coherence**: Researchers have demonstrated significant advances in quantum error correction and the extension of quantum coherence lifetimes. These developments are key to building more reliable and efficient quantum computing systems【9†source】【11†source】.

5. **Industrial and Academic Collaborations**: Institutions like Caltech and companies like Broadcom have announced partnerships to advance quantum science research, signaling a growing trend of collaboration between academia and industry in this field【14†source】. Furthermore, the UTSA co-hosted Quantum Collaborative Summit highlights the national priority given to training and research in quantum information science【21†source】.

6. **Investment and Market Viability**: There has been substantial investment in quantum computing by both private companies and governments globally. This financial support is pivotal for overcoming the challenges in scaling quantum computing systems and achieving market viability【12†source】【18†source】.

7. **Integration with AI and High-Performance Computing**: Microsoft's integration of Copilot with Azure Quantum Elements represents a trend of merging AI models and high-performance computing with quantum computing. This integration aims to improve simulation accuracy in material and pharmaceutical research, indicating the potential of quantum computing in diverse scientific fields【22†source】【16†source】.

8. **Future Prospects and Challenges**: Despite these advancements, the field faces challenges like individual qubit control at scale, cooling power requirements, and manufacturability issues. The journey towards a fully functioning, large-scale quantum computer is still ongoing, with the exact timeline being difficult to predict【12†source】.

Overall, 2023 has been a year of substantial progress in quantum computing, marked by technological advancements, increased collaboration, and significant investment. The field is moving towards overcoming existing challenges and unlocking the potential of quantum computing in various applications.
